# Comparing PM_2.5_, respirable dust, and total dust fractions using real-time and gravimetric samples in an exposure chamber study

**DOI:** 10.1016/j.heliyon.2023.e16127

**Published:** 2023-05-23

**Authors:** Therese Nitter Moazami, Kristin v Hirsch Svendsen, Morten Buhagen, Rikke Bramming Jørgensen

**Affiliations:** a Department of Industrial Economics and Technology Management (IØT), Norwegian University of Science and Technology (NTNU), 7491, Trondheim, Norway; b Department of Occupational Medicine, St. Olav's University Hospital, 7006, Trondheim, Norway

**Keywords:** DustTrak DRX, Real-time sensors, Occupational exposure, Gravimetric samples

## Abstract

Using an exposure chamber, we investigate the precision of the DustTrak DRX monitor by comparing its results to those obtained from taking traditional gravimetric samples of two stone minerals commonly used in asphalt and lactose powder. We also discuss the possibility of using real-time monitors such as DustTrak DRX for occupational exposure monitoring purposes.

The results are based on 19 days of experiment, each day with measurements collected over 4 h. Compared to the gravimetric samples, the DustTrak DRX overestimated the PM_2.5_ and respirable dust concentrations, while it underestimated the total dust concentration by a factor of nearly two. However, the ratios, being done for more than one material, between the DustTrak DRX and the gravimetric sample readings varied daily and across the different exposure materials.

Real-time sensors have the potential to excel at identifying exposure sources, evaluating the measured control efficiency, visualizing variations in exposure to motivate workers, and contributing to the identification of measures to be implemented to reduce exposure. For total dust, a correction factor of at *least two* should be used to bring its readings up to those for the corresponding gravimetric samples. Also, if the DustTrak DRX is used in the initial profiling of occupational exposure, the exposure could be considered acceptable if the readings are well below the occupational exposure limit (OELs) after correction. If the DustTrak DRX readings, after correction, is close to, or above, the accepted exposure concentrations, more thorough approaches would be required to validate the exposure.

## Highlights

• Real-time monitors are becoming popular for assessing occupational exposure.
• Real-time monitors offer many benefits over the traditional gravimetric samples.
• Compared to gravimetric samples, the DustTrak DRX underestimated total dust.
• Compared to gravimetric samples, the DustTrak DRX overestimated smaller particles.
• A sheet with correction factors for common dusts can make real-time monitors valid.

## 1 Introduction

Occupational exposure to dust is prevalent in various industries, such as mining, welding, construction, metal casting, food production, stonework, and woodwork. Dust is an unspecific term encompassing various materials, which can differ significantly in size, shape, and density. Depending on its aerodynamic diameter, a particle can be deposited in various regions of the respiratory tract. In occupational settings, particle fractions are often assigned to inhalable, thoracic, or respirable dust [1]. While the inhalable fraction refers to the mass fraction of total airborne particles aspirated through the nose and/or mouth (<100 μm in aerodynamic diameter); [2], the thoracic and respirable fractions refer to the mass fractions of inhaled particles passing through the larynx and unciliated airways, respectively [3,4]. Additionally, a “total dust” sample is often used to provide a dust fraction between the inhalable and thoracic fractions [5].

To assess occupational exposure to total and respirable dust, gravimetric samples are routinely used. Although it is the European and U.S. reference sampling method for measuring various dust fractions [6], gravimetric sampling typically requires a long time, especially when there are low exposure concentrations. While a long sampling time may be appropriate when an exposure assessment aims to verify compliance with 8-h occupational exposure limit (OEL) values, gravimetric samples are incapable of addressing temporal and spatial heterogeneities in exposure and providing information on peak exposures [7,8]. Traditional gravimetric sampling and analysis are also expensive.

Over the years, the methods and terms used in occupational epidemiology have shifted from general to specific because new or unknown risks are challenging to detect [11]. When the prevalence of a disease - the number of exposed people- or the exposure concentrations are low, even a tiny confounding variable or measurement error might prevent the discovery of an association between exposure and disease. Therefore, exposure estimates must be accurate and reliable [12] as well as optimized so that the study design, sampling strategy/collection of data, and methods used in exposure analyses reduce the chance of estimation error [11]. Low-level exposures must also be assessed to ensure workplace attractiveness, maintain workers' well-being, and improve productivity, workability, and performance [13].

Real-time instruments are valuable in terms of providing information about particle or gas concentration variations observed during exposure days [9] and are becoming more popular [10]. Additionally, real-time instruments enable the collection of high-resolution, low-cost measurements for quickly identifying peak, short-term, and low exposure concentrations; linking exposure concentrations to emission sources; and obtaining a better understanding of the within-day exposure variation [14,15]. Additionally, the exposure-time curve obtained from real-time logging instruments can be used to visualize workers' exposure, perhaps motivating the workers to identify where measures to reduce exposure are needed.

DustTrak DRX Aerosol Monitors enable real-time monitoring of various particle fractions at high temporal resolutions [9]. These instruments combine photometric measurements of the particle cloud and optical sizing of single particles in a visual system and measure different particle size fractions, total dust, respirable dust, and particulate matter (PM_2.5_, PM_10_, and PM_1_) [16]. Light-scattering technologies have become increasingly popular for occupational monitoring purposes [[17], [18], [19]]. However, previous studies have shown that these technologies are not as accurate as gravimetric sampling [20], as they are sensitive to the size distribution, shape, angle, and composition of particles [9]. Specifically, light-scattering technologies have been found to overestimate the PM_2.5_ fraction compared to gravimetric sampling [[21], [22], [23], [24]], while underestimating the PM_10_ concentration [25]. In one previous study, the DustTrak DRX underestimated the coarse fractions by approximately 20% in samples collected in a desert environment [26]. In another study, where samples of PM_10_ were collected in pig houses and poultry yards, the DustTrak DRX underestimated the gravimetric samples by nearly a factor of two [25].

The road minerals quartz diorite and rhomb porphyry are commonly used in Norwegian asphalt. Exposure to these stone minerals is prevalent in occupational and public health, as asphalt wear significantly contributes to local air pollution [27,28]. In this paper, we investigate the precision of the DustTrak DRX monitor by comparing its results with those obtained using traditional gravimetric sampling of each of the three exposure materials and discuss the possibility of using real-time monitors such as DustTrak DRX for occupational exposure monitoring purposes.

## 2 Materials and methods

In this paper, dust samples collected for a randomized double-blinded controlled exposure chamber study are examined. The study involved 24 healthy, non-smoking young adults (volunteers), who were exposed to quartz diorite, rhomb porphyry, and lactose powder. Details on the exposure chamber and aerosol generation, along with the observed health effects of exposure to the stone aggregates on lung inflammation, lung function, and blood coagulability, can be found elsewhere [[29], [30], [31]]. To control the exposure in the chamber, personal gravimetric samples of respirable dust and stationary gravimetric samples of total dust, thoracic dust, PM_2.5_, and respiratory dust were collected during each 4-h exposure session. Additionally, stationary real-time data samples of total dust, respirable dust, PM_10_, PM_2.5_, and PM_1_ were collected continuously during each exposure session using the DustTrak TSI DRX 8533.

### 2.1 The exposure chamber

**Fig. 1:**
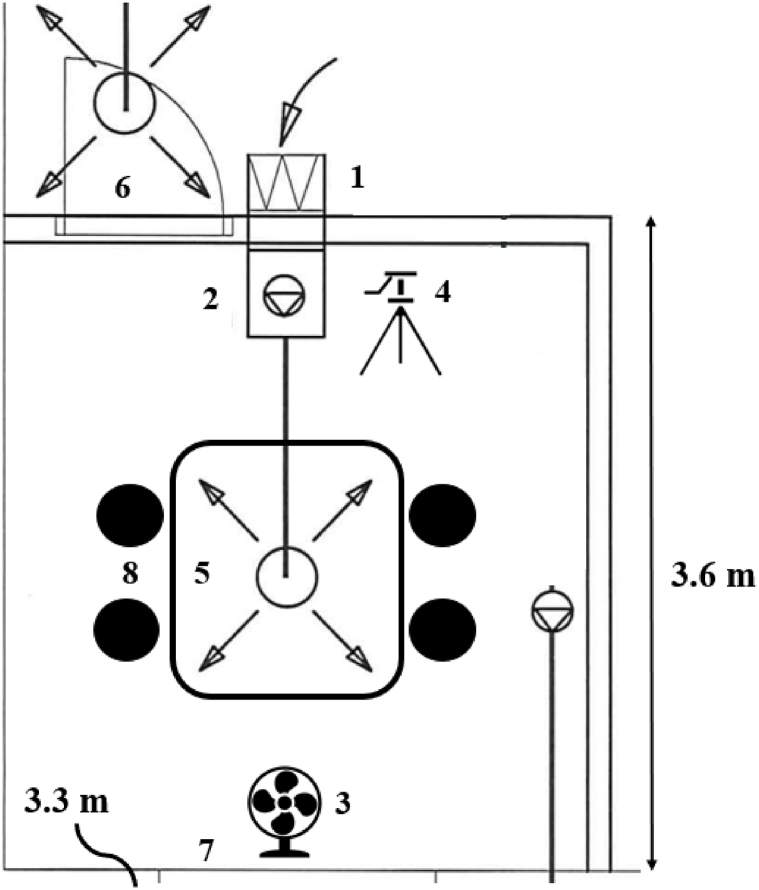
The exposure chamber. Stationary samples were collected from the test stand, while personal samples were collected from each of the four people seated at the table. Symbols: 1 = HEPA filter, 2 = pump, 3 = table fan, 4 = test stand, 5 = circular diffusor, 6 = entrance door, 7 = window facing outdoor, 8 = chair.

The exposure concentration in the chamber varied slightly, and to account for the variation due to location, the volunteers changed seats every hour. The air entering the chamber was taken from the room next to the exposure chamber and filtered through a HEPA filter (see Fig. 1, symbol 1) to make sure that the particles measured in the chamber were only from the dust generated into the exposure chamber. The floor, walls and roof in the exposure chamber consisted to smooth surfaces which made it easy to clean in between each exposure session. Using DustTrak DRX, the particle concentrations in the room were measured before each exposure session started to make sure that the room had been sufficiently cleaned. All participants received written and oral information about the study and signed an informed consent before accepted as participants in the clinical trial. This study was approved by the Regional Ethics Committee (REK) in Trondheim, Norway (approval no. 260381) and was conducted in accordance with the guidelines in the Research Ethics Act, the Health Research Act, and the Personal Data Act.

### 2.2 Gravimetric and real-time data samples

Gravimetric and real-time (DustTrak DRX) samples were collected from the same location at a test stand in the exposure chamber (see Fig. 1), and the particle distribution is therefore considered identical for all the stationary samples collected in this study. Additionally, personal samples of respirable dust were collected from each of the volunteers.

The personal respirable dust samples were measured using cyclones (SKC aluminum respirable cyclones) equipped with cassettes (SKC SureSeal three-piece 37 mm) containing polyvinyl chloride (PVC) filters (SKC PVC filter 37 mm 5.0 μm). The filters were mounted in the breathing zones of the volunteers during the exposure session and coupled to a sampling pump (SKC AirChek 3000), which was set to deliver a flow rate of 2.5 L/min for 4 h.

The stationary gravimetric samples were collected on the filters using sampling pumps (Casella Tuff personal air samplers). For total dust, an SKC Sure Seal three-piece 37 mm cassette with an airflow of 2.0 L/min was used, and for PM_2.5_, the PEM 2.5 μm cassette (MSP corporation, 5910 Rice Creek Parkwayu, Suite 3000. Shoreview, MN; USA) with an airflow of 2.0 L/min was used. The respirable and thoracic fractions were collected using the SKC Sure Seal three-piece 37 mm cassette equipped with cyclones, i.e., the SKC aluminum respirable cyclone with an airflow of 2.5 L/min and the BGI GK 2.69 cyclone with an airflow of 1.6 L/min, respectively.

Additionally, from the same location at the stationary test stand, real-time data samples of total dust, PM_10_, respirable dust, PM_2.5_, and PM_1_ were measured continuously during exposure using the DustTrak DRX, which was calibrated with standard ISO 12103-1 A1 test dust (Arizona road dust) at the factory. Arizona road dust is an inhomogeneous mixture of different stone minerals [32] and is considered to be representative of coarse mineral dust. Pre- and post-airflow calibration was performed on all sampling pumps and the DustTrak DRX (Mettler Instruments AG, Greifensee, Switzerland) for each exposure session.

Also attached to the test stand was a Scanning Mobility Particle Sizer (SMPS) (TSI Model 3938) for the continuous monitoring of ultrafine particles (UFPs) and submicron particles in the size range 9.63–437.1 nm. The SMPS was fitted with a 0.071-cm impactor nozzle, and the flowrate through the instrument was 1.5 L/min.

### 2.3 Calibration of the dust to be piped into the exposure chamber

The TSI 3410 Dust Aerosol Generator was used to force quartz diorite and rhomb porphyry into the exposure chamber, and the Aerosol Generator TSI 3400 was used to add lactose powder. Before each exposure session started, the belt speed and feed mass combination for the Dust Aerosol Generator and chamber ventilation was set so as to not exceed the Norwegian 8-h OELs for total dust (10 mg/m^3^) and respirable dust (5 mg/m^3^) exposure, respectively [29]. In particular, an air change rate (ACH) of 3.0/h was used during quartz diorite and rhomb porphyry exposure, and a belt speed between 10 and 11% was employed after the respirable dust fraction was recorded by the DustTrak DRX.

While quartz diorite and rhomb porphyry are dry powders, lactose powder contains moisture (5–6%), causing these particles to aggregate after being piped into the chamber and making it impossible for the same high concentrations to be achieved during exposure. To keep the concentrations of lactose powder as high as possible, a lower ACH (2.06/h) was used during exposure, along with a bed purge of 80 and a belt speed between 20 and 30%. It was, however, challenging to record above 3–4 mg/m^3^ on the DustTrak DRX during lactose powder exposure.

### 2.4 Exposure dust composition

The mineral compositions of quartz diorite and rhomb porphyry were analyzed using X-ray Diffraction (XDR), and the results are shown in Table 1. Both aggregates were crushed using the standardized Los Angeles (L.A.) method (European Committee for Standardisation 1998, EN 1097–2 annex A).

**Table 1:** The mineral compositions (in %) of quartz diorite and rhomb porphyry.

Material	Quartz	K-feldspar	Plagioclase	Amphibole	Calcium	Epidote	Chlorite	Muscovite
Quartz diorite	27.5	12.0	32.0	–	–	15.0	10.5	3.0
Rhomb porphyry	4.5	31.0	47.0	3.0	2.0	–	5.5	7.0

**Fig. 2:**
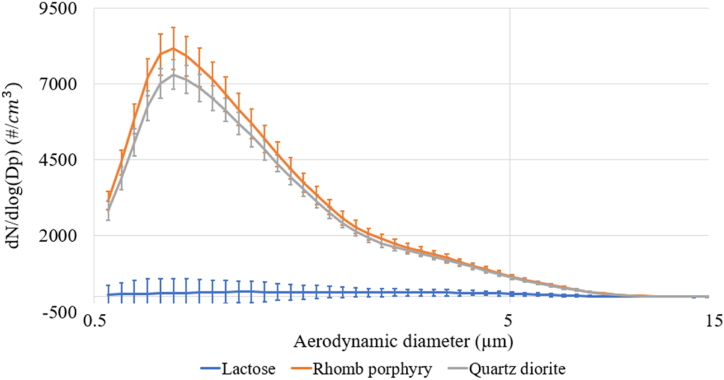
Normalized concentrations and standard deviation of the three dust types.

The size distributions of the three different exposure materials were analyzed using the Aerodynamic Particle Sizer® (APS) from TSI (Model 3321) and measured from the same location at the test stand as the other stationary samples. The geometric mean diameters and geometric standard deviations (GSDs; given in parentheses) for quartz diorite, rhomb porphyry, and lactose were 1.20 (1.77), 1.18 (1.76), and 1.19 (1.96), respectively. The normalized concentrations and standard deviations for the three different dust types are shown in Fig. 2. Quartz diorite and rhomb porphyry had similar particle size distributions, each with a dominating mode of 0.78 μm. The lactose powder had a different particle size distribution, with a dominating mode of 1.38 μm. According to the manufacturer, Arizona road dust has a density of 2.65 g/cm^3^, a GSD of 1.57, and a geometric mass mean diameter of 2.12 μm [16].

### 2.5 Statistical analyses

The concentrations measured using the DustTrak DRX were averaged over the 4 h of each exposure session to be compared with the gravimetric samples. According to the Shapiro Wilk test, only the personal gravimetric respirable dust samples and DustTrak DRX measurements of PM_1_ followed a normal distribution. Therefore, along with its 95% confidence interval (CI), the median was used for descriptive purposes. ANOVA with a Bonferroni correction was used for the dust fractions following a normal distribution. For the remaining dust fractions, exposure comparisons between the exposure materials were made using the Kruskal Wallis test. Spearman's correlation was used to measure nonparametric bivariate correlations.

The ratios between the results obtained via gravimetrical sampling and the DustTrak DRX were calculated by dividing the PM fraction measured gravimetrically by the PM fraction measured using the DustTrak DRX, see Formula 1. For descriptive purposes, the median ratios, along their 95% CIs, are included in the results.

(1) $$R=\frac{{PM}_{grav}}{{PM}_{DT}}$$

The samples collected gravimetrically and using DustTrak DRX were paired separately for each volunteer and exposure material for comparisons using the Wilcoxon signed-rank test. Statistical significance was set as p < 0.05 (two-tailed). All statistical analyses were performed using SPSS version 28.

### 2.6 Quality control

The DustTrak DRX was thoroughly cleaned before each exposure session, and the internal filter (37 mm glass fiber filters) were changed between each exposure session. Additionally, a calibration to zero was performed before each exposure session using a HEPA filter. The filters were weighed in the same room both before and after sampling and under the same environmental conditions. A filter blank was weighed together with the experimental filters for the gravimetric samples.

## 3 Results and discussion

Across the 19 days of the experiment, one gravimetric sample of total dust was rejected due to cassette leakage. In addition, the stationary gravimetric samples of respirable dust were only collected on two days of exposure to quartz diorite, three days of exposure to rhomb porphyry, and one day of exposure to lactose powder.

### 3.1 Comparison between gravimetric and real-time measurements

Table 2 shows the median concentrations measured for each exposure material and their 95% CIs (in parentheses). As shown in the table, the concentrations of all particle fractions measured during exposure to lactose powder were significantly lower than those achieved during exposures to quartz diorite and rhomb porphyry. The moisture content in the lactose powder likely caused this difference, as it most possibly encouraged particle aggregation.

**Table 2:** Medians (95% CI) for various dust fractions measured gravimetrically and using DustTrak DRX.

Measurement	Quartz diorite	Rhomb porphyry	Lactose powder
Number of sessions (each 4 h long) (mg/m^3^)	6	6	7
Total dust (gravimetric)^b^ (mg/m^3^)	20.8 (15.8–24.1)	21.9 (20.2–25.9)	5.7 (4.3–6.2)
Total dust (DustTrak DRX)^b^ (mg/m^3^)	11.4 (11.1–12.1)	14.6 (13.6–14.8)	3.4 (3.3–3.6)
Thoracic dust (gravimetric)^b^ (mg/m^3^)	13.9 (13.3–16.7)	17.0 (16.1–18.8)	2.3 (1.2–2.7)
PM_10_ (DustTrak DRX)^b^ (mg/m^3^)	11.4 (10.9–11.9)	14.5 (13.5–14.5)	3.1 (3.0–3.3)
Respirable personal (gravimetric)^a^ (mg/m^3^)	5.4 (4.7–6.1)	5.7 (4.9–6.4)	0.3 (0.3–0.5)
Respirable dust (DustTrak DRX)^b^ (mg/m^3^)	8.5 (8.4–8.7)	10.1 (9.4–10.3)	1.4 (1.4–1.5)
Respirable dust (gravimetric)^b^ (mg/m^3^)	7.7 (7.6–7.9)	8.4 (8.4–9.1)	0.7***
PM_2.5_ (gravimetric)^b^ (mg/m^3^)	4.3 (4.2–4.6)	5.2 (4.2–13.7)	1.2 (0.9–1.2)
PM_2.5_ (DustTrak DRX)^b^ (mg/m^3^)	6.8 (6.8–6.8)	7.7 (7.1–8.2)	1.1 (1.1–1.2)
PM_1_ (DustTrak DRX)^b^ (mg/m^3^)	5.9 (5.8–6.0)	6.7 (6.1–7.2)	1.1 (1.0–1.1)
UFP (SMPS) (number/cm^3^)	1726 (1464–1816)	2343 (2208–4785)	678 (371–783)
Submicrometer particles (SMPS) (number/cm^3^	14491 (13954–15015)	21287 (18309–26425)	337 (334–372)

a Personal sample.

b Stationary sample, ***Samples were collected on only one of the exposure days.

Overall, slightly higher concentrations were achieved in the chamber during exposure to rhomb porphyry compared to quartz diorite. According to the Kruskal Wallis test, the difference observed in the concentrations of personal respirable dust (gravimetric), thoracal dust (gravimetric), and concentrations of total dust (gravimetric) were not statistically significantly different when comparing quartz diorite and rhomb porphyry, which means that the concentrations of these particle fractions cannot be considered to be different during exposure to quartz diorite and rhomb porphyry. However, the concentrations of the remaining particle fractions, namely, the stationary PM_2.5,_ and respirable fractions, were measured gravimetrically, and the samples collected using DustTrak DRX differed for the two stone minerals.

According to the Wilcoxon signed-rank test, a significant difference was observed between the total dust samples collected gravimetrically and using DustTrak DRX for all three exposure materials (see Table 3). In our study, the median ratios (95% CIs) observed between gravimetric samples and DustTrak DRX for total dust were 1.9 (1.5–2.1), 1.8 (1.3–1.8), and 1.7 (1.4–1.8) for quartz diorite, rhomb porphyry, and lactose powder, respectively, meaning that DustTrak DRX underestimated the samples collected gravimetrically by a factor of nearly two. Also, note that the 95% CI of the ratio for total dust shows that the ratio varies from day to day, meaning that multiple parallel samples taken via DustTrak DRX and gravimetry are needed to establish a correction factor between the two sampling strategies.

**Table 3:** Summary of Wilcoxon signed-rank test results.

Dust material		Test statistics		
		N	Z	P
Quartz diorite	Respirable gravimetric^b^– Respirable personal gravimetric^a^	8	−2.52	0.01
	Respirable DustTrak DRX^b^– Respirable personal gravimetric^a^	23	−4.20	<0.01
	PM_2.5_ DustTrak DRX^b^ – PM_2.5_ Gravimetric^b^	23	−1.58	**0.11**
	Total dust DustTrak DRX^b^ – Total dust Gravimetric^b^	23	−3.82	<0.01
	Respirable DustTrak DRX^b^ – Respirable Gravimetric^b^	8	−2.59	0.01
	Thoracic Gravimetric^b^ – PM_10_ DustTrak DRX^b^	23	−4.20	<0.01
Rhomb porphyry	Respirable gravimetric^b^– Respirable personal gravimetric^a^	11	−2.93	0.00
	Respirable DustTrak DRX^b^– Respirable personal gravimetric^a^	23	−4.20	<0.01
	PM_2.5_ DustTrak DRX^b^ – PM_2.5_ Gravimetric^b^	23	−1.83	**0.07**
	Total dust DustTrak DRX^b^ – Total dust Gravimetric^b^	23	−4.02	<0.01
	Respirable DustTrak DRX^b^ – Respirable Gravimetric^b^	11	−2.97	0.00
	Thoracic Gravimetric^b^ – PM_10_ DustTrak DRX^b^	23	−2.92	<0.01
Lactose	Respirable gravimetric^b^– Respirable personal gravimetric^a^	4	−1.83	**0.07**
	Respirable DustTrak DRX^b^– Respirable personal gravimetric^a^	24	−4.29	<0.01
	PM_2.5_ DustTrak DRX^b^ – PM_2.5_ Gravimetric^b^	24	−0.32	**0.75**
	Total dust DustTrak DRX^b^ – Total dust Gravimetric^b^	24	−4.29	<0.01
	Respirable DustTrak DRX^b^ – Respirable Gravimetric^b^	4	−1.86	**0.06**
	Thoracic Gravimetric^b^ – PM_10_ DustTrak DRX^b^	23	−3.41	0.00

a Personal sample.

b Stationary sample. Bold p-values indicate insignificant differences between samples.

Although the definitions of the thoracic fraction and PM_10_ are slightly different, these particle fractions have a 50% cut-off at 10 μm and are, to some extent, equivalent [2]. When paired, the concentration of the thoracic particles measured gravimetrically and the PM_10_ concentration measured using DustTrak DRX were statistically different (see Table 3). However, the median ratios between the thoracic fraction and PM_10_ were 1.3 (1.2–1.4), 1.2 (1.0–1.3), and 0.7 (0.4–0.9) for quartz diorite, rhomb porphyry, and lactose powder, respectively, meaning that these measured concentrations were fairly similar across the two sampling methods.

When comparing the respirable gravimetric samples obtained stationarily versus personally, median ratios of 1.6 (1.2–2.0), 1.5 (1.3–1.6), and 2.0 (1.5–2.2) for quartz diorite, rhomb porphyry, and lactose powder were found, respectively, meaning that higher concentrations were measured stationarily compared to personally. The higher concentrations collected stationarily compared to personally are likely caused by the placement of the test stand, as the test stand was closer to the dust dispersal inlet than the table where the volunteers were seated.

For the respirable fraction, the ratios between the gravimetric (personal) samples and the DustTrak DRX (stationary) samples were 0.6 (0.5–0.8), 0.6 (0.5–0.7), and 0.3 (0.2–0.3) for quartz diorite, rhomb porphyry, and lactose powder, respectively, meaning that the DustTrak DRX overestimated the respirable fraction compared to the personal respirable samples collected gravimetrically. According to the Wilcoxon signed-rank test, the differences observed between the gravimetric and DustTrak DRX samples were statistically significant for all three exposure materials (see Table 3).

Most previous studies comparing gravimetric and DustTrak DRX samples have focused on PM_2.5_. In one study, gravimetric and DustTrak DRX samples of PM_2.5_ were compared after assessing exposure to residual fuel ash and welding fumes, for which the geometric mean PM_2.5_ concentrations were almost identical. A Spearman rank correlation coefficient of 0.68 was obtained between the two types of measurement strategies. The authors concluded that aerosol particle characteristics may affect the relationship between the gravimetric and DustTrak DRX measurements and that recalibration for the specific aerosol, as recommended by the factory, may be necessary to obtain valid results [33]. In another study, gravimetric samples and samples collected using DustTrak DRX were compared in a desert environment; the fine fraction was overestimated by a factor of approximately two [26]. This result corresponds with the results of another study, where gravimetric and real-time samples of PM_2.5_ from moving trucks were compared, in which the authors found a Spearman's correlation between the two measurements of 0.63. The DustTrak DRX measurements exceed the gravimetric measurements by a factor of two [8]. An overestimation of two (2.07 for summer, and 2.02 winter) for the DustTrak DRX compared to the gravimetric samples was also found in another study, where 12-h samples of PM_2.5_ were collected in a test chamber and field. However, after the DustTrak DRX response was corrected, the Pearson's correlation between the two measurement strategies increased to 0.87 and 0.81 in the summer and winter, respectively [34]. The same conclusion was reached in another paper where two direct-reading instruments were compared with a federal reference method for PM_2.5_, i.e., it was concluded that the accuracies of the direct-reading instruments could be improved via statistical adjustments [35].

In our study, the median ratios between the PM_2.5_ measured gravimetrically and using DustTrak DRX were 0.6 (0.6–0.8), 0.7 (0.6–1.9), and 1.0 (1.0–1.0) for quartz diorite, rhomb porphyry, and lactose, respectively, meaning that the DustTrak DRX overestimated the concentrations of quartz diorite and rhomb porphyry. In contrast, the two methods measured similar concentrations of lactose powder. Judging by the 95% CI, the ratios between the samples collected gravimetrically and using DustTrak DRX differed according to exposure day, especially for rhomb porphyry, which had ratios ranging from 0.6 to 1.9. Nevertheless, when the PM_2.5_ samples collected gravimetrically and using DustTrak DRX were paired (see Table 3), no significant differences were observed in these fractions for the exposure materials.

Note that the photometric calibration factor settings on DustTrak DRX were not updated for the specific dust type for each exposure day. Instead, the ratios observed between the gravimetric and DustTrak DRX samples were calculated using formula 1. In addition, before the experiment started, the dust generator was adjusted so that the exposure concentrations measured gravimetrically would not exceed the 8-h OELs for total dust and respirable dust. The corresponding concentrations on the DustTrak DRX were noted several times for each dust type so that the DustTrak DRX could be adjusted accordingly during the experiment.

While a significant correlation was observed between the real-time readings and gravimetric samples collected for total dust, PM_2.5_, and personal respirable samples during exposure to quartz diorite (see Table 4), no consistency was observed for total dust and personal respirable particles measured stationarily and gravimetrically during exposure to rhomb porphyry and lactose powder, despite quartz diorite and rhomb porphyry having the same size distribution (see Fig. 2). In a previous study, the precision of the DustTrak DRX readings for PM_10_ declined for higher concentrations of PM_10_ (>200 μg/m^3^) [26], which might explain the low precision observed in our study, as the exposure concentrations in the chamber were high. Lower PM concentrations may be more relevant when measuring outdoor or indoor air pollution, while higher PM concentrations are usually of concern in occupational environments where dust exposure occurs.

**Table 4:** Spearman correlations between average real-time readings and gravimetric sample readings.

Material	Total dust^a^ vs. total dust^b^	PM_2.5_^a^ vs. PM_2.5_^b^	Resp personal^a^ vs. Resp stat^b^	Resp personal^a^ vs. Resp stat^a^	PM_10_^b^ vs. Thoracic^a^
Quartz diorite	0.64**	−0.54**	−0.73*	0.87**	0.58**
Rhomb porphyry	−0.41	0.25	0.29	−0.78**	0.39*
Lactose powder	0.14	0.52**	0.13	–	0.51**

**significant at the 0.05 level, * significant at the 0.10 level.

a Gravimetric.

b DustTrak DRX.

### 3.2 Using the DustTrak DRX for occupational exposure monitoring purposes

One of the main challenges of real-time monitors, such as those in the DustTrak DRX family, is size, making them less suitable for assessing personal exposure. For assessing occupational exposure, personal samples are always preferred to stationary samples, which, in most cases, cannot be considered to be representative of personal exposure. Furthermore, it is essential to recognize that real-time monitors, such as Split 2, Dataram, and Sidepak, have previously been found to underestimate the concentration of inhalable dust while overestimating the respirable particle fraction. For these instruments, a decreasing precision has been found with increasing particle size [36], possibly caused by their varying responses to the properties of the dust. Also, seasonal variations may affect the relationship between the measurements taken by gravimetric and real-time monitors [33]. In our study, the dust content supplied to the chamber was known and constant for each of the three dust types. In real-life occupational settings, the dust in a room may come from various sources, making it challenging to use correction factors. Additionally, the correlation between the DustTrak DRX and the gravimetric samples in this study varied daily, indicating that any potential correction factor should be investigated using several calibration tests. Also, the software of the DustTrak DRX does not allow for the calibration of all the dust fractions measured. Considering the deviation between DustTrak DRX and the gravimetric samples vary with the particle fraction, calibrating for a single particle fraction may not make the DustTrak readings more accurate.

Real-time sensors are becoming increasingly popular, and despite their disadvantages, usage will probably increase in the coming years. A data sheet with correction factors for common types of dust could be developed to match the readings by various types of sensor technology up with gravimetric readings. However, individual correction sheets would be necessary for each instrument family, as the particle concentrations measured with varying instruments may not be comparable, even when calibrated with the same dust. In our study, the DustTrak DRX overestimated the PM_2.5_ and respirable dust concentrations compared to the gravimetric sampling done simultaneously from the same test stand. The total dust concentration was underestimated by a factor of nearly two when comparing the DustTrak DRX measurements with those obtained using gravimetric sampling, suggesting that, for the particle fractions below total dust, the DustTrak DRX can be used to give a conservative estimate of the occupational exposure. This finding aligns with previous studies in which the DustTrak DRX overestimated the PM_2.5_ fraction. For total dust, however, the DustTrak DRX sample readings should be multiplied by a factor of two when used to assess occupational exposure. If the DustTrak DRX total dust readings are well below the OELs, even after correction, the exposure could perhaps be considered acceptable. If the DustTrak DRX samples are unacceptably high after correction, then gravimetric samples should be collected. In such a case, the DustTrak DRX could provide an important initial evaluation of occupational exposure.

## 4 Conclusions

Real-time sensors have the potential to excel in identifying sources contributing to the exposure, evaluating the measured control efficiency, visualizing variations in exposure to motivate workers, and contributing to determining which measures should be implemented to reduce exposure. However, the DustTrak DRX underestimated total dust exposure, and a correction factor of at least two should be used to help its measurements correspond to those obtained using gravimetric samples. For smaller particle fractions, however, the DustTrak DRX overestimated exposure. If the DustTrak DRX is corrected for readings that deviate from gravimetric sample readings, this instrument will provide valuable information in an initial evaluation of occupational exposure.

## Author contribution statement

Therese Nitter Moazami: Analyzed and interpreted the data; Contributed reagents, materials, analysis tools or data; Wrote the paper. Kristin v Hirsch Svendsen, Morten Buhagen, Rikke Jørgensen: Conceived and designed the experiments; Performed the experiments; Wrote the paper.

## Data availability statement

The datasets generated and analyzed during the current study are available from the corresponding author on reasonable request.

## Funding statement

This work was funded by the Research Council of Norway [grant numbers 260381].

## Additional information

No additional information is available for this paper.

## Declaration of competing interest

The authors declare that they have no known competing financial interests or personal relationships that could have appeared to influence the work reported in this paper.
